# Neonatal transport practices and effectiveness of the use of low‐cost interventions on outcomes of transported neonates in Sub‐Saharan Africa: A systematic review and narrative synthesis

**DOI:** 10.1002/hsr2.1938

**Published:** 2024-03-07

**Authors:** Emmanuel Okai, Frankie Fair, Hora Soltani

**Affiliations:** ^1^ Department of Paediatrics, School of Medical Sciences, College of Health and Allied Sciences University of Cape Coast Cape Coast Ghana; ^2^ Department of Nursing and Midwifery, College of Health, Wellbeing and Life Sciences Sheffield Hallam University Sheffield UK

**Keywords:** Kangaroo mother care, low‐cost interventions, neonates, Sub‐Saharan Africa, transport

## Abstract

**Background and Aims:**

Neonatal deaths contribute significantly to under‐5 mortality worldwide with Sub‐Saharan Africa (SSA) alone accounting for 43% of global newborn deaths. Significant challenges in the region's health systems evidenced by huge disparities in health facility deliveries and poor planning for preterm births are major contributors to the high neonatal mortality. Many neonates in the region are delivered in suboptimal conditions and require transportation to facilities equipped for specialized care. This review describes neonatal transport across the subregion, focusing on low‐cost interventions employed.

**Methods:**

We conducted a systematic review of studies on neonatal transport in SSA followed by a narrative synthesis. A search in the databases CINAHL, EMBASE, MEDLINE, Web of Science, African Index Medicus, and Google Scholar was performed from inception to March 2023. Two authors reviewed the full texts of relevant studies to determine eligibility for inclusion which was subsequently cross‐checked by a third reviewer using a random 30% overlay. The quality of the included studies was assessed using the Mixed Methods Appraisal Tool.

**Results:**

A total of 20 studies were included in this review involving 11,895 neonates from 10 countries. All studies evaluated the transfer of neonates into referral centers from the peripheries. Most neonates were transferred by public transport (*n* = 12), mostly in the arms of caregivers with little communication between referring facilities. Studies reporting on ambulance transfers reported pervasive inadequacies in both human resources and transport equipment. No study reported on the use of Kangaroo mother care (KMC) in the transfer process.

**Conclusions:**

The neonatal transport system across the SSA region is poorly planned, poorly resourced, and executed with little communication between facilities. Using cost‐effective measures like KMC and improved training of community health workers may be key to improving the outcomes of transported neonates.

**Study question**: What is the evidence on neonatal transport in Sub‐Saharan Africa? What low‐cost interventions are employed in neonatal transport in Sub‐Saharan Africa to improve morbidity and mortality in transported neonates?

**What's already known**: A poorly structured transport process in developing countries is a major contributor to increased neonatal mortality rates. WHO recommended low‐cost interventions including Kangaroo mother care improve outcomes in neonates stabilized within neonatal units in developing countries. Differing interventions to improve thermal and physiologic stability have been studied in other world regions.

**What this study adds**: As the only systematic review on neonatal transport practices in Sub‐Saharan Africa, this study provides context‐specific current evidence on studies performed in a subregion that has significant disparities compared to other regions classified as developing or Low‐middle income countries.

## 1 BACKGROUND

Significant progress has been made in child survival since the institution of the Millennium Development Goals (MDGs), over 2 decades ago. However, despite increased facility delivery, which was a focus of the MDG, neonatal mortality in low and low–middle‐income countries (LMIC) remains high.

Even though over 62 countries were able to achieve MDG 4, the target was missed globally with the under‐5 mortality rate (U5MR) reducing by 53% over 25‐year period (1990–2015) instead of the targeted 75%.^1^

Globally, U5MR fell from 90.6 deaths per 1000 live births in 1990 to 42.5 per 1000 live births in 2015,^1^ significantly below the MDG target. In percentage terms, the decline in neonatal mortality has been slower than that of postneonatal under‐5 mortality: reducing by 47% compared with 58% globally. As a result, the portion of neonatal deaths among overall under‐5 deaths increased from 40% in 1990 to 45% in 2015,^2^ with several regions' resource‐limited settings exceeding a mortality rate of more than 50%.^3^

The Sustainable Development Goals (SDG), a follow‐up from the MDGs has an aim for all countries to achieve a U5MR of 25 or fewer deaths per 1000 livebirths by 2030 (SDG target 3.2.1) and a neonatal mortality rate (NMR) of 12 or fewer deaths per 1000 livebirths by 2030.^4^ About halfway into the SDG era, the global U5MR is estimated to have decreased by 59% to 37.7% deaths per 1000 live births in 2019 and NMR reduced by just over half from 36.6% in 1990 to 17.5% deaths per 1000 live births in 2019.^5^ This translates into a drop in the total number of deaths from 5.0 million in 1990 to 2.4 million in 2019.^5^

Sub‐Saharan Africa (SSA) is currently made up of 48 out of the 52 countries on the African continent and is geographically located south of the Sahara Desert,^6^ with an estimated total population of approximately 1.18 billion.^7^ It has differing statistics, economically and in health service delivery compared to other regions considered LMIC. Approximately, 40% of the world's population are extremely poor and live below the US$1.90‐a‐day poverty line. SSA accounts for 66% of the global extremely poor.^8^

SSA and Southern Asia alone accounted for 80% of the global neonatal deaths in 2019.^9^ Two‐thirds of these occurred on the first day of life and almost three‐quarters within the first week. The challenge to survive encountered by the neonate, therefore, starts at the place of delivery, with a risk of death up to 20 times higher in SSA and Southern Asia compared to high‐income countries.^5^

Many newborn deaths are a consequence of a lack of contact with the health system or a lack of transfer systems to access higher‐level facilities.^10^, ^11^, ^12^ Unfortunately, in SSA, sick newborns in need of transfer to established specialized neonatal centers remain at risk of adverse outcomes because of under‐resourced peripheral delivery facilities, lack of coordinated neonatal transport service, and frequently unsafe transfer practices. Prompt attention to appropriate referral of sick newborns might therefore significantly contribute to a reduction in the high mortality occurring on the day of birth.^13^

Challenges in every sector of the health system in SSA and most LMICs regarding newborn care including the absence of well‐established ambulance services lend themselves to the need to explore the possibility of employing cost‐effective but evidence‐based, life‐saving procedures in the neonatal transfer process. The results from such an exercise would inform technical guidelines for use by healthcare professionals, facilities, and organizations responsible for setting health system priorities and policies surrounding the transport of neonates in LMICs. The use of Kangaroo mother care (KMC) and other alternative methods of thermal stability for neonatal transport is not well studied in SSA, yet this has been one of the areas proposed for future research in several published studies on neonatal transport.

KMC is a practical, effective, and safe substitute for conventional neonatal care in preterm and low birthweight (LBW) infants mainly in resource‐limited countries. It involves strapping the baby upright to the mother's chest in skin‐to‐skin contact. World Health Organization defines KMC with four components: early, continuous, and prolonged skin‐to‐skin contact between the newborn and mother, exclusive breastfeeding, early discharge from the health facility, and close follow‐up at home.^14^ Previous studies have reported its benefits in reducing the risk of morbidity and mortality among LBW infants.^15^ The skin‐to‐skin component alone has also been reported to be associated with improved breastfeeding, cardiorespiratory stability, and improved responses to procedural pain in randomized controlled studies.^16^, ^17^

### 1.1 Rationale

While a review of neonatal transport in developing countries has been undertaken,^11^ the field is advancing necessitating an updated review. The use of KMC for the care of stable newborns within neonatal units in resource‐limited settings has been reported but there is a dearth of information on its use in neonatal transport. This study aimed to systematically review the literature on neonatal transport in SSA to explore all components including the use of cost‐effective procedures.

## 2 METHODS

A systematic review was undertaken according to the prospectively developed review protocol which conformed to Preferred Reporting Items for Systematic Reviews and Meta‐analysis (PRISMA) guidelines.^18^ The review is registered with PROSPERO (registration number CRD42022352401).

### 2.1 Literature search

A systematic literature search was conducted from inception to March 2023 of the following databases: CINAHL, EMBASE, MEDLINE, Web of Science, African Index Medicus, and Google Scholar. A combination of subject headings and keyword searches about neonates, transport or transfer, transport interventions, and all Sub‐Saharan African countries was used. These were tailored to each database. The full list of search terms is available in (Appendix S1).

There were no restrictions placed on the date or language of the publication. However, the studies included were restricted to only those from SSA. Bibliographies of retrieved articles were reviewed for additional articles which meet the inclusion criteria. All selected articles were imported into RefWorks, ProQuest reference manager, and independently assessed by reviewers using the inclusion/exclusion criteria.

### 2.2 Inclusion criteria

All peer‐reviewed publications on neonatal transport, describing transport modalities/processes and outcomes within SSA were assessed.

### 2.3 Exclusion criteria

Case reports, protocols, editorials, commentaries, or studies on neonatal transport that were not published in SSA. For studies with uncertainty about meeting the inclusion criteria, or where relevant data could not be extracted from the study report, the authors were contacted via email for additional data or clarification. If this was not forthcoming the study was excluded. In the case of studies of mixed samples where only a proportion of the study population meets the inclusion criteria (e.g., only a proportion of the transported infants are neonates, or communication was partly about the maternal prelabour transfer), the study was included if relevant data could be extracted.

### 2.4 Definitions

A neonate is defined as a newborn infant with a chronological age of 28 days or less without regard to the postmenstrual age at birth.

A caregiver is defined as the individual who accompanies the neonate during transport, as identified by the study, and could be a family member, such as a mother, father, aunt, grandmother, or friend.

Pretransport, intratransport, and posttransport interventions refer to all interventions instituted to improve the outcome of referred neonates. These include training in the communication of medical personnel, interfacility prereferral communication, interventions that aim to refine or upgrade already existing methods of transport, and the introduction of new methods or equipment for transport and methods to improve physiologic stability.

Facility type was categorized as either primary, secondary, or tertiary health centers as identified by each study. Primary health centers included public and private maternity units, health centers, and health facilities with in‐patient care as defined by individual studies based on the country's health structure.

Healthcare providers include hospital/facility administrators, paramedics, and medically trained providers such as doctors, midwives, and nurses.

### 2.5 Data extraction

Articles retrieved using the search strategy were initially screened by title and abstract against the inclusion criteria by E. O. and checked by H. S. and F. F. with any disagreement over potential article relevance resolved through discussion.

Eligible papers were read in full text independently by E. O. and F. F. for inclusion and E. O. extracted study characteristics and outcome data from the included studies using a predefined data extraction form (Excel spreadsheet) which was subsequently checked for accuracy by H. S. or F. F. Papers which met the inclusion criteria at the abstract stage, but where a full‐text article could not be obtained even after contact with authors were included if enough data could be extracted from the abstract. Data extracted were summarized to include, authors, country of study, the study aims and objectives, research methods/design, participants, information on the transport/transfer process, main findings, quality appraisal score, and study limitations. Summary characteristics were kept broad due to the vast methodological differences within and between the studies. For qualitative studies, directly reported participant data (e.g., verbatim quotations or scores), and author interpretations were extracted and analyzed separately in order not to distort the contextual basis required to understand barriers and enablers of an efficient neonatal transportation system for the region.

### 2.6 Quality appraisal

All reviewers independently assessed the 20 full‐text papers using the Mixed Methods Appraisal Tool (MMAT), Version 2018.^19^ Articles were assessed using the criteria within the tool that was appropriate for the study design; quantitative (randomized; nonrandomized: descriptive), qualitative, or mixed‐methods design. Any disagreements were discussed within the team. No studies were excluded based on their quality but commented on in the narrative synthesis.

### 2.7 Data summary and synthesis

The heterogeneity among the included studies, even for studies with similar methodologies made it taxing for combining quantitative data using meta‐analysis, or a meta‐synthesis for qualitative data. The general framework and specific tools outlined in the Economic and Social Research Council Guidance on the Conduct of Narrative Synthesis in Systematic Reviews^20^ were applied.

We, therefore, proceeded to combine studies by summarizing their descriptive statistics followed by a textual narrative synthesis as opposed to a thematic synthesis. A textual synthesis held more potential to describe the gaps in the literature and to make transparent the heterogeneity between the studies as well as make the context and study characteristics of each study clearer.

To minimize the inherent selection bias of facility‐based surveys regarding their population sample as well as minimizing bias in synthesizing studies, we have attempted to describe all outcomes and findings regardless of their statistical significance.

## 3 RESULTS

**Figure 1:**
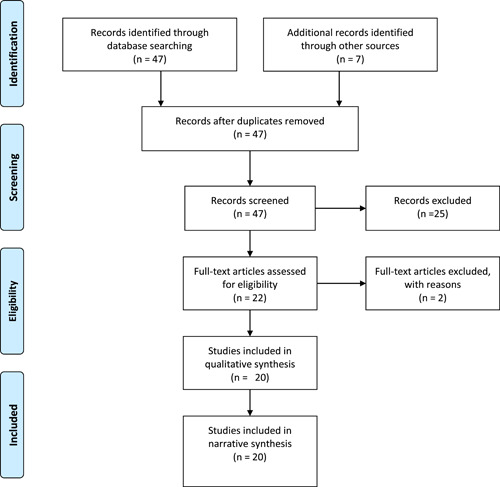
Preferred Reporting Items for Systematic Reviews and Meta‐analysis flowchart.^23^

Figure 1 gives a PRISMA flow diagram of the study search and selection process.^18^ Forty‐seven publications were identified, and seven articles were identified through hand‐searching the bibliographies of identified studies. Duplicates were removed leaving 47 papers that were screened against the inclusion/exclusion criteria by their titles and abstracts. A total of 22 studies were reviewed in full text. Out of these, two articles were excluded based on the exclusion criteria: The first is a registry‐based study that focused on sociodemographic factors, pregnancy complications, and neonatal factors predicting admission to the neonatal unit without any distinction of outborn neonates^21^ and the second study involved mother‐baby pairs with data analysis mainly focussing on the mothers.^22^ A total of 20 articles met our inclusion criteria and were included in the final review and analysis.

### 3.1 Study characteristics

Table 1 provides a summary of study characteristics. Five studies (25%) were conducted in South Africa,^24^, ^25^, ^26^, ^27^, ^28^ four (20%) were conducted in Nigeria,^12^, ^29^, ^30^, ^31^ two each (10%) were conducted in Ethiopia^32^, ^33^ Ghana,^34^, ^35^ and Senegal,^36^, ^37^ and one each (5%) from Cameroon,^38^ Congo,^39^ Guinea,^40^ Mali,^41^ and Uganda.^42^ There were no randomized controlled studies. Two studies were qualitative^27^, ^32^ with the rest being descriptive cross‐sectional studies: 11 retrospective and 7 prospective.

**Table 1:** Study characteristics.

Study ID (year)	Country	Study design	Aims and objectives	Dates of data collection	Participant demographics	Level of transfer	Conclusion	Study limitations
1. Roux et al.^24^ (1989)	South Africa	Descriptive Cross‐sectional Quantitative Retrospective	Study of quality of interhospital transport of ill neonates by ambulances	May to June 1987	15 neonates 9 full terms, 6 preterms 10 neonates weighed < 1 kg	Interhospital transfer within the Witwatersrand province	Lack of equipment, especially in private ambulances increases risk. Staff development and control by a local national body recommended.	Small sample size Short study period
2. Njokanma and Fagbule^29^ (1994)	Nigeria	Descriptive Cross‐sectional Retrospective Quantitative	Review of the outcomes of neonates < 2.5 kg transferred for tertiary care	Retrospective March 1989 to December 1990 Prospective January to August 1991	103 neonates (59 male [M], 44 female [F]) 77.7% preterm 55.3% <1.5 kg 44.7% 1.5–2.5 kg	Transfers of outborn neonates (from home maternity units and primary centers) into a tertiary service	Resuscitation with emphasis on thermoregulation at delivery centers and adequate neonatal transport may improve outcomes in high‐risk neonates.	No comparison was made to neonates delivered in the same unit who received similar care
3. Pieper et al.^28^ (1994)	South Africa	Descriptive Cross‐sectional Retrospective Quantitative	Description of the mode of transport and outcomes of transported neonates to NICU	January to September 1992	52 neonates, 29 M:23 F Gestational age 28–43 weeks	All neonates referred to a level 3 neonatal intensive care unit	High survival rate in transported infants, highest mortality in the asphyxia‐related meconium aspiration syndrome and infants transported by ground ambulance.	No stated sampling methods The target population is not stated
4. Ndiaye et al.^36^ (2003)	Senegal	Descriptive Cross‐sectional Retrospective Quantitative	Description of factors associated with mortality of low‐birth‐weight neonates transferred into a tertiary Hospital	January 1998 to December 1999	180 LBW new‐borns Mean weight 1452.5 ± 432 g	Community and peripheral centers into tertiary care	Prevention of LBW and improved collaboration between obstetricians and pediatricians is needed to improve the outcome of transferred neonates.	No comparison made with neonates of different weights better appreciate the associated factors
5. Enweronu‐Laryea et al.^34^ (2008)	Ghana	Descriptive Cross‐sectional Retrospective Quantitative	Retrospective cohort comparing birth weight‐specific survival and referral pattern of newborns requiring intensive care before and after improvement of facilities.	2007	Admissions into NICU during the study period (2078 before and 1815 after refurbishment) out of which (178 vs. 287) were referred	Transfer from public and private health facilities to a tertiary center	Survival of referred newborns <2.5 kg improved from 56% (before) to 68% (after) Improvements in NICU facilities without attendant improvements in emergency obstetric services and neonatal referral systems are not enough to achieve the desired reduction in neonatal mortality.	Lack of data on transport characteristics of transferred neonates resulting in hypothesized exposure–effect relationship.
6. Dicko et al.^41^ (2010)	Mali	Descriptive Cross‐Sectional Retrospective	Description of problems associated with neonates transferred into a tertiary neonatal service	October 2006 to January 2007	760 referred newborns out of 1072 neonatal admissions. M: 57.7% F: 42.2% Preterm: 33.6% LBW: 43.6%	Transfer from community centers, maternity units and peripheral hospitals	91.6% of the cases were referred on the very day of their birth. Prematurity, perinatal anoxia respiratory distress were the main reasons for transfer Maternal illiteracy, LBW, and outborn birth were factors related to neonatal mortality.	Difficult to determine how representative the sample size was. The definition of hypothermia is not standard
7. De Vries et al.^25^ (2011)	South Africa	Descriptive Cross‐Sectional Retrospective	A retrospective evaluation of the impact of a dedicated obstetric and neonatal transport service on transport times within an urban setting	January to December 2005 and January to December 2008	All ambulance calls were coded for flying squad service during the period of study. 3257 flying squad calls out of a total of 46,074 in 2005 and 4865 flying squad calls out of a total of 65,885 in 2008	State‐run ambulance service providing essential prehospital emergency care to the population of the Western Cape of South Africa	Significant improvement between 2005 and 2008 in all incidents evaluated. Performance improved from 11.7% to 46.6% of all incidents dispatched within 4 min. The program resulted in a reduction in total prehospital time from 177 to 128 min.	1. Absence of patient outcome measures Teams may have executed transfers more efficiently, but the appropriateness of the dispatch or the quality of the clinical management cannot be determined. 2. Potential investigator bias
8. Nalwadda et al.^42^ (2013)	Uganda	Descriptive Cross‐sectional Retrospective	Assessment of the timely compliance (within 24 h) with newborn referrals made by community health workers (CHWs), and its determinants	September 2009 and August 2011	724 newborns were referred by CHWs of whom 700 were successfully traced. 51% males, 49% female. 56% were delivered at a health facility. The mean age of mothers is 27 years, 93% married	Community to hospital referrals	The newborn being sick, being born to a younger mother and a reminder visit by the CHW to a referred newborn were strong predictors of newborn referral compliance.	
9. Nsibande et al.^27^ (2013)	South Africa	Qualitative Cross‐sectional study, Interviews were conducted using a structured questionnaire	Substudy to assess the effectiveness of the CHW referral system (in a bigger community randomized trial) by describing CHW referral completion rates as well as mothers' healthcare‐seeking practices	June 2008 to June 2010	148 Referred sick Neonates. The mean age of mothers −24 years. Most had attended some high school; the majority were single and unemployed. 39% of mothers were HIV positive, 56% negative, and 5 (4%) had missing status.	Community to hospital	High compliance (95%) with CHW referral of sick babies in an urban setting. This suggests that CHWs can play a significant role, within community outreach teams, to improve newborn health and reduce child mortality.	
10. Katamea et al.^39^ (2014)	Congo	Descriptive cross‐sectional prospective	To study the frequency of extrahospital neonatal transfer to the neonatology unit and identify the risk factors associated with mortality.	January 2012 to December 2012	All neonates less than 7 days transferred and cared for until discharge. 150 transferred neonates out of 1412 admissions 12.9% F: 58, M: 92 LBW 64%	Maternity units and other health centers to a tertiary facility	Mortality was significantly related to maternal profession (saleswoman or worker), high level of education, gestational age <37 weeks dystocia vaginal delivery, male sex, birth weight <1.5 kg, and prematurity	Selection of cases discussed but no mention of criteria which led to the exclusion of 33 out of the 183 factors
11. Nlend et al.^38^ (2016)	Cameroon	Descriptive, Cross‐Sectional Prospective	To describe the transport modalities (means of transport, the reason for transfer, transfer delay, number of detours, the prevalence of hypothermia and the neonatal mortality rate among referred newborns	October 2014 to January 2015	73 newborns referred during the study period. 1/73 was born outside the city and 72 were born in Yaoundé, (22%) were born within the health district of the referral facility‐tertiary level or equivalent (33%), a lower‐level structure type district hospital in (20%), a first‐level health center in (47%). 29/73(40%) preterm	33% from the tertiary level or equivalent 20% from a lower‐level structure such as a district hospital & 47% from a first‐level health center	Transfers of sick newborns are associated with poor prognosis because of an erratic itinerary, with an increased risk of hypothermia and death.	Small sample size Lack of use of methods of accessing metabolic stability for long journeys
12. Abdulraheem et al.^30^ (2016) and Olukemi et al.^31^ (2020)	Nigeria	Descriptive Cross‐Sectional Prospective	To evaluate the modes of transport, pre‐ and intratransport care of neonates referred to a tertiary neonatal care service	August 2012 to February 2013	401 neonates presenting to the emergency room of a tertiary hospital 87.3% singletons. 3.5% triplets, 9.2% twins 67.1% term 31.4% preterm and 1.5% postterm neonates 41.4% females 31.4% preterm	General or private hospitals home/mission/traditional birth attendants’, primary health centers & other tertiary centers. 1 neonate was found abandoned by the roadside	No aspect of referral was adequate from communication to preparation/stabilization for transport and the actual transport itself.	
13. Ashokcoomar and Naidoo^43^ (2016)	South Africa	Descriptive Cross‐Sectional Prospective	To investigate delays in the transfer of neonates between healthcare facilities and to detect any adverse events encountered during neonatal transfer.	December 2011 to January 2012	120 interhealthcare facility transfers of neonates 62.2% of transfers were by frontline ambulances; 29.2% by the obstetric unit; 1.7% by the planned patient transport vehicles. 76.7% of the neonates were preterm, 85% LBW	77.5% were referred from hospitals and 22.5% were referred from primary healthcare clinics 57.5% were primary transfers for a specialized or higher level of care, 42.5% were return transfers	47.3% delays in dispatch >3 min due to ambulance nonavailability Delays in 47.3% due to no paramedic available or 32.4% of transfers where a paramedic was deemed to be required, no personnel to support the neonatal transfer on 4.7% of occasions in 15.5% no neonatal transfer equipment available.	The total study population is not known. The study was over 2 months
14. Faye et al.^37^ (2016)	Senegal	Descriptive Cross‐Sectional Prospective	To evaluate the characteristics of neonates transferred between 8 selected district hospitals and health centers in the Dakar region and 3 university referral hospitals	August 2013 to January 2014	130 transfers. 76.1% from peripheral centers to university hospitals and 23.8% between university hospitals. M:F 1.6:1. Preterm 37.8% LBW 33.1% 67% of transfers were for families with a low or average socioeconomic level. 42% of newborns were referred <24 h and 52% within 48 h. 8.7% were home deliveries	Between 8 selected district hospitals and health centers in the Dakar region and 3 university hospitals	Neonatal transfers are carried out in poor conditions in Dakar. The implementation of an effective neonatal transport system organized around regional. Perinatal networks are a priority.	
15. Accorsi et al.^33^ (2017)	Ethiopia	Descriptive Cross‐Sectional Prospective	To evaluate the cost‐effectiveness of an ambulance‐based referral system for emergency obstetric and neonatal care in rural Ethiopia	January to April 2015	111 obstetric cases referred to the hospital via ambulance 5% were transferred from the village via the Health center to the hospital, and 95% were referred from the health. Center to the hospital	Community to public/private hospital	The service was undoubtedly and possibly effective for the mother and/or the newborn in 8% and 24% of cases, respectively (a total of 36 cases, corresponding to 32%). It was possibly effective for an additional 22 women and 23 newborns. Corresponding to 336 years saved. The cost per year of life saved was US$24.7 which is below the benchmarks of 150 and US$30 that define attractive and very attractive interventions.	The study exclusively focussed on survival and did not consider the quality of life and disability which may also be of relevance. The judgment on effectiveness remains theoretical and subjective
16. Sory et al.^40^ (2019)	Guinea	Descriptive Cross‐Sectional Prospective	To identify risk factors and study the causes of neonatal mortality of LBW newborns referred to a tertiary service	March to August 2015	250 referred newborns 56.4% male, 47.2% Female. Gestational age <28 weeks 28% 28–32 weeks 8.3% 32–37 weeks 6% >37 weeks 1.2% Birth weight <1 kg 9.2% 1–1.5 kg 49.3% 1.5–2.5 kg 41.6% 40.4% referred from maternity hospitals in university hospitals	All referrals to a tertiary service	45.3% of mortality in neonates admitted aged <24 h compared to 59.3% for those admitted >24 h. Risk factors associated with neonatal mortality were gravidity, parity, occupational activities requiring physical effort, birth weight, and prematurity.	The physiologic stability of neonates on arrival was not examined
17. Teklu et al.^32^	Ethiopia	Qualitative	To assess the barriers to the effective functioning of the referral system for preterm, LBW, and sick newborns across the primary health care units	December 2017 to February 2018	Mothers of premature, LBW, or sick newborns, mean age 20–29 years and primiparous, Obstetric and newborn care providers 56%—midwives and 40% nurses Facility administrators in the public health care system 74% from health centers, 26%—from primary and referral hospitals	Referrals across the 3 tiers of the public health system	Gaps and barriers in the newborn referral system were identified in 3 areas: Inadequate transport and uncoordinated referral/feedback communication between referral facilities and providers; availability of, and adherence to newborn referral protocols; and family reluctance or refusal of newborn referral. because of the expectation of infant death despite referral, and patient costs related to referral.	Mothers in one of the sample sites were not involved in the in‐depth interviews due to geography and difficulty in contacting them given the limited resources available to the authors
18. Tette et al.^35^ (2020)	Ghana	Descriptive Cross‐Sectional Retrospective	To evaluate the mode of transport, distances traveled, condition on arrival and outcome of referred newborns	Regional (level 2) Hospital January to October 2018 District (level 1) hospital January to June 2018	153 caregivers/parents of referred newborns. Case notes reviews Male 57.5% Female 42.5% Preterm 17.0% LBW 13.7%	Referrals of outborn neonates to a regional and district hospital	The median time spent at the first referral health facility by the patients who died was four times that of those who survived. A greater proportion of neonates who died traveled by public transport (bus) and arrived in unstable conditions. Findings suggest there may have been delays initiating transfers or better stabilization required before or during the transfer.	
19. Okonkwo et al.^12^ (2020)	Nigeria	Descriptive Cross‐sectional Retrospective	To assess the transfer process, modes of transportation, distances covered to access care, condition on arrival, and outcomes of newborns admitted to a tertiary hospital	February 2015 to January 2016	Responsible persons for 115 babies Preterm 32.2% LBW 37.3% Male 63.5% Female 36.5%	Transfers from the community, maternity units, and peripheral hospitals into a tertiary service	Poorly developed neonatal transport system with a systematic lack of hospital‐to‐hospital communication from referral to accepting hospital and feedback to the referring facility. Transfers often occur without appropriate staffing, monitoring, and simple interventions.	Lack of use of standard definitions of measures of clinical stability

Abbreviations: HIV, human immunodeficiency virus; LBW, low birthweight; NICU, neonatal intensive care unit.

The focus of the studies was varied. Three studies were community‐based surveys,^27^, ^32^, ^42^ two studies evaluated the quality/effectiveness of a dedicated neonatal ambulance service,^24^, ^25^ one study evaluated barriers to an effective neonatal transport service,^32^ one study evaluated the cost‐effectiveness of a dedicated ambulance service^33^ and the rest (13) were facility‐based surveys.

The majority (*n* = 18/20) of the studies were published after 2000 with only two studies published between 1989 and 1994. Twelve of the studies was published after the last review of neonatal transport in developing countries.^11^ All studies evaluated the transfer of neonates into referral centers from the peripheries.

The study populations were homogeneous in terms of age, as the ages of all the study participants enrolled were less than 1 month. The subgroup categories within studies however differed for example in gestational age, birthweight, and severity of illness as summarized in Table 1.

The included studies identified a total of 11,895 neonates who were either transferred into tertiary care from the peripheries or benefited from interhospital transport. Out of these, 848 neonates were included in two nonfacility‐based studies which analyzed referrals by only community health workers into hospitals.

Although included studies had a varied focus, the majority focused on the description of the reasons for transfer and outcomes of neonates transported into the hospital (*n* = 14).^12^, ^27^, ^29^, ^30^, ^31^, ^34^, ^35^, ^36^, ^37^, ^38^, ^39^, ^40^, ^41^, ^43^

### 3.2 Quality assessment

The quality of studies using the MMAT assessment is shown in (Appendix S2). All studies met the MMAT screening criteria questions. The two qualitative studies were of good quality with clearly defined research questions, sampling methods, and clear links between data collected and inferences. The quantitative studies were of varied quality. Most studies addressed the research question well but a few studies either had unclear aims^38^ or the stated aim was not addressed in the final analysis.^36^

Most quantitative studies described the target population well and used appropriate measurements. 15/20 studies were judged to have a sample that was representative of the target population, in 5/20 studies it was not possible to tell if the sample was representative. In sampling their participants, only one stated they used convenience sampling, however, a closer review of the studies suggested that most studies used convenience sampling. Most studies did not comment on any consideration about how study findings or the extent of missing data introduced biases. The discussion segments of some studies were limited and most studies often limited analyses to descriptive statistics on comparison groups and *χ* ^2^ or *t* tests to elucidate differences in outcomes.

### 3.3 Synthesis of results

A neonatal transport service has several key components: Human resources, vehicles, and equipment, communication and family support, documentation and quality assurance, and feedback to referring units. Included studies varied greatly in their focus on the individual aspects of the transport service. These have been incorporated into four overarching themes including “mode of transport,” “support during transport,” “referral pathway and communication,” and “neonatal care and access within health facilities.”

### 3.4 Mode of transport: Organized versus self‐transport

As shown in Table 2, 16 studies either examined or commented on the mode of transport of neonates in the subregion. The mode of transport and neonatal outcomes were similar across studies regardless of study quality. Five studies examined regionalized/organized ambulance transport services using ground transport,^4^, ^24^, ^43^ private ground ambulances,^33^ fixed‐wing helicopters, and ground ambulances.^28^ Eight studies identified public transport as the main mode of transport with the majority (7/8) being transferred by taxis^12^, ^34^, ^35^, ^37^, ^38^, ^40^, ^41^ and private vehicles.^30^ In one study, a comment is made about 10% of the transfers being made by ambulance but the other modes of transfer are not analyzed.^36^ Transfers on foot was the maximal mode of transport in three studies,^39^, ^42^ with one reporting as high as more than 30%.^40^ Reported average distances for many transfers were <80 km but up to 200 km in helicopter transfers^28^ (Table 2). The reported time of travel was however considerable ranging from 15 min to 48 h.^35^, ^38^, ^43^ The reported average delay before admission to a referral center was 3.5 h with a maximum of 3 days.^37^

**Table 2:** Mode and organization of transport.

Study ID (year)	Organization	Mode of transport	Distance traveled	Travel time	Timing of referral	Vehicles and equipment	Human resources	Communication, documentation, and consent	Pretransport stabilization/monitoring during transport	Feedback to referring units
1. Roux et al.^24^ (1989) South Africa	Provincial	Neonatal ICU transfers 4 provincial and 11 private ambulance transfers	20–80 km 6 transfers <32 km	Not analyzed	No reported delays	Ground ambulances Inadequate Equipment in private ambulances	Paramedics A gap in training noted especially for private ambulances	Good verbal communication. Written communication less complete especially in private ambulances. Poor documentation of the maternal history, delivery details, and treatment before and during transport. No written consent from parents	All neonates had good Apgar scores and had vital signs checked before and after transport. No comment was made on monitoring during transport	Not analyzed
2. Njokanma and Fagbule^29^ (1994) Nigeria		Not analyzed	Not analyzed	Not analyzed	Not analyzed	Not analyzed	Not analyzed	Not analyzed	Not analyzed	Not analyzed
3. Pieper et al.^28^ (1994) South Africa	Public Health Service	Aircraft and ground ambulances	Up to 200 km	Not analyzed	Not analyzed	Fixed‐wing aircraft, helicopters, specially equipped ambulances	Doctors for helicopters, specially trained ambulance personnel	All transfers were planned	Not analyzed	Not analyzed
4. Ndiaye et al.^36^ (2003) Senegal	Public Health Service	Ambulance used in 10% of transfers	Not analyzed	Not analyzed	Not analyzed	Not analyzed	Not analyzed	Not analyzed	Not analyzed	Not analyzed
5. Enweronu‐Laryea et al.^34^ (2008) Ghana	Public Health Service	Mostly by taxi	Not analyzed	Not analyzed	Not analyzed	Not analyzed	Not analyzed	Not analyzed	Not analyzed	Not analyzed
6. Dicko et al.^41^ (2010) Mali	Public Health Service	58.6%—public transport, ambulance—17.4%	Not analyzed	Not analyzed	Not analyzed	All the newborns are transferred into the arms of a member of their family.	Health personnel accompanied newborns in ambulance transfers	No advanced communication from the referring facility in all cases. The referring personnel was a doctor in 70.3% of cases and a midwife/nurse/caregiver/matron in 29.7% of cases.	Not analyzed	Not analyzed
7. De Vries et al.^25^ (2011) South Africa	Public Health Service Regionalized Perinatal Service	State‐run ambulance service	Not analyzed	Mean total prehospital time improved from 177 min in 2005 to 128 min in 2008	The mean time to dispatch is 78 min	Equipped ambulances	Ambulance crew. Personnel not analyzed	Prearranged transfers	Not analyzed	Not analyzed
8. Nalwadda et al.^42^ (2013) Uganda	Public Health Service. Health Demographic Surveillance Site	Walking was the most common means used by 47% of the caretakers	The mean distance traveled was 2 km	Not analyzed	87% of newborns referred during the first week of life at a median age of 3 days	Vehicle transport not analyzed	Caretakers accompanied by transported neonates	Not analyzed	Not analyzed	Not analyzed
9. Nsibande et al.^27^ (2013) South Africa	Public Health Service	Not analyzed	Not analyzed	Not analyzed	Not analyzed	Not analyzed	Not analyzed	Not analyzed	Not Analyzed	Only16% of health workers gave written feedback on the outcome of the referral to the referring CHW
10. Katamea et al.^39^ (2014) Congo	Public health service	73.3% transferred on foot. the rest 26.7% by public transport out of which taxis—22% None by ambulance	Not analyzed	Not analyzed	All newborns <7 days 43.3% were transferred more than 24 h after birth	Public transport 26.7% Equipment not analyzed	Newborns transferred wrapped and carried in the hands of a family member	In no case was the service informed in advance of the transfer	Not analyzed	
11. Nlend et al.^38^ (2016) Cameroon	Public Health Service	The majority of transfers provided by the family mainly use taxis and personal vehicles, with 6.8% of ambulance transfers.	Not analyzed	The average transfer time was 17 h	60% transferred <6 h of life, 22% within the first 2 h of life. In >50%, a transfer to another hospital was done before admission		The nurse accompanied in 10/73 13.6% of transfers	Comments made about the poor chain of information is a major component of neonatal transfer and the cause of several detours	Not analyzed	Not analyzed
12. Abdulraheem et al.^30^ (2016) and Olukemi et al. (2020)^31^	Public Health Service	4% by ambulances 43.9% taxis 28.9% motorcycle 9% tricycles 1.7% commercial buses 10% on foot from referral centers	61.1% traveled within a 10 km radius. 6.9% traveled between 10 km and 20 km and 2.0% traveled between 20 and 80 km	Not analyzed	85% of the neonates presented in the first week of life. 33.7% within the first 12 h of life, 12.5% at 12–23 h, 9.0% at 24–48 h of life	4% transferred in hospital‐owned ambulances. Ambulances were nonfunctional in 2.3% of health facilities. One ambulance was suitable to carry transport incubators. 62.8% of neonates were transported in the “arms of the caregivers” and 35.4% strapped to the caregivers' backs. 1 (0.2%) had a well‐wrapped hot water bottle applied. None used KMC.	25.0% by both mothers and fathers, 31.4% by mothers, 38.4% by fathers 5.5% by the relatives without either of the parents. Only 7% of neonates were accompanied by a nurse or other health professional	42% of the babies had referral letters while 17.3% were verbally referred from these hospitals without any letters given to them. Only 51% of the referral letters had complete information. There was no prior communication from any of the referring centers	Pretransport care included resuscitation (18.2%), intravenous fluid/feeding (24.4%) and supplemental oxygen (14.0%). 8.7% (11/126) of preterm compared with 2.9% (8/275) of term neonates died in the course of transportation	No feedback from the referral center with any of the referring centers.
13. Ashokcoomar and Naidoo^43^ (2016) South Africa	Public Health Service	The local public ambulance provider	Not analyzed	Meantime 3 h 49 min Range 0 h 55 min to 10 h 34 min	Not analyzed	On 15.5% of occasions, neonatal transfer equipment was not available.	No paramedic was available for 32.4% of transfers where a paramedic was deemed to be required	Not analyzed	Not analyzed	Not analyzed
14. Faye et al.^37^ (2016) Senegal	Public Health Service	An ambulance was used in 30% of transfers and medicalized in 3 transfers; the urban taxi was the most used mode of transport at 45.4%	Not analyzed	Not analyzed	The mean delay before admission to a referral center was 3.5 h (maximum of 3 days) 53.8% had visited at least two referral centers	Only 3 medicalized ambulances were used. Equipment not analyzed	72.3% of transfers were accompanied only by their parents without any health personnel	Referral centers were notified in 35 cases (26.9%). In 83.9% of cases) a reference document was attached: these were essentially handwritten letters in 77.7%	39.2% had pretransfer resuscitation. 43.1% had bag and mask ventilation 13% had external cardiac massage. Resuscitation was provided by a pediatrician in 2 cases. 37.7% had received no care before the referral	Not analyzed
15. Accorsi et al.^33^ (2017) Ethiopia	Public Health Service partly financed and coordinated by an NGO	One ambulance supplied by an NGO	2.5 –47 km	Not analyzed	Not analyzed	An ambulance was equipped with a single couch but could carry up to three other seated persons if needed	Drivers with no specific training who received instructions. from healthcare professionals of the hospital or the health Centers. No healthcare professionals accompanied the driver	The call for the ambulance service was made by the pregnant woman or her family member through cell phone to the ambulance driver's cell phone or a fixed phone located at the hospital	Not analyzed	Not analyzed
16. Sory et al.^40^ (2019) Guinea	Public Health Service	52.8% arrived by taxi, 33.2% on foot, 8.8% by other forms of transport and 4.8% by ambulance	Not analyzed	Not analyzed	89.2% referred < 24 h. 10.8% > 24 h	4.8% Ambulance transfers Equipment not analyzed	Not analyzed	74.4% were referred by physicians. Means of communication not analyzed	Not analyzed	Not analyzed
17. Teklu et al.^32^ (2020) Ethiopia	Public Health service	Not analyzed	Not analyzed	Not analyzed	Not analyzed	Not analyzed	Not analyzed	Noted as one of the major barriers to effective neonatal transport	Not analyzed	Noted as a major barrier
18. Tette et al.^35^ Ghana	Public Health Service	Taxi 34.0% Bus 29.4% Motor cycless 26.8% Walked 5.2% Tricycle 2% Ambulance 0.7% Unspecified 1.3%	The mean distance of 23.1 km 0.93–91.15 km) for survivors and 2.69–75.66 km for those that died. 28.3% traveled <10.00 km, 26.3% 10.00–19.99 km, 42.8% 20.00–79.99 km and 4 (2.6%) traveled >80 km	Median time 1 h 20 min, Range 15 min to 48 h	9.2% < 1 day old, 40.5% < 7 days old 50.3% > 7 days old Time from onset of symptoms to arrival at the hospital 25.5% < 24 h 74.5% > 24 h	Not analyzed	Child's father 44.4% Other family member 26.1% Friend 9.1% Not indicated 1.3% One patient accompanied by a nurse	Not analyzed	Not analyzed	Not analyzed
19. Okonkwo et al.^12^ (2020), Nigeria	Public Health Service	Tricycle 0.9%, Foot 0.9% Ambulance 3.5% Bus 13.9% Car 80.9%	10–20 km 54.7% Unknown 14.8% 20–50 km 14.8% >100 km 7.9% ≤5–10 km 7.9%	Not analyzed	Not analyzed	None of the babies were transported in transport incubators, nor given skin‐to‐skin care	Relative 88.7% Nurse 7.8% Doctor 1.7%, Paramedic 0.9% Pharmacist 0.9%	Most cases (75%) arrived from peripheral hospitals without referral notes or prior contact with the receiving hospitals Parents were just verbally instructed to go to the receiving hospital	There was no organized pre‐ or intratransfer. stabilization or medical interventions	Not Analyzed but comment made about poor feedback to referring units

Abbreviations: ICU, intensive care unit; KMC, Kangaroo mother care; NGO, nongovernmental organization.

*Support during transport*: This is subdivided into (a) Use of equipment, KMC, or other low‐cost interventions for transport and (b) human resources and family support:

(a) *Use of equipment, KMC, or other low‐cost interventions for transport*: In no study was KMC employed during transport. In five studies using ambulance transport, there was reported inadequate equipment during the transfer^24^, ^30^, ^32^, ^41^, ^43^ and one study reported on an adapted ambulance with no equipment.^33^ One of these reported one neonate transferred with a hot water bottle to maintain thermal stability.^30^ In all other studies, transfers were made with babies in the caregivers' arms. No study examined any innovative models for transport or used illness severity scores (TRIPS, TOPS, SNAP‐II, SNAPPE‐II) in assessment, triage, and prediction of mortality^44^, ^45^ (Table 2).
(b) *Human resources and family support*: The five studies which examined a regionalized/organized transport service involved trained paramedics to accompany the transfer. In all the other remaining studies, transported neonates were accompanied by mothers or other family members. A health worker accompanied a proportion of the transfers in three studies – 13% with nurses,^38^ 1.7% with a doctor and nurse,^12^ and 7% with a nurse or other health professional^30^ (Table 2).

*Referral pathway and communication*: All studies reported a lack of standard referral pathways with critical deficiencies in communication before, during, and after transport with the majority reporting no communication between referring units (Table 2).

### 3.5 Neonatal care and access within health facilities

Eleven studies reported on the outcome of newborns transported into tertiary neonatal units for continued care with consistently high mortality in outborn transported neonates. Only 1 out of these 11 studies reported mortalities of <10%,^35^ 8 studies between 20% and 32%.^12^, ^28^, ^30^, ^34^, ^37^, ^38^, ^39^, ^41^ In one study the mortality was 46.8%,^40^ with the highest mortality being 63.5%.^29^ Two studies with high mortalities reported significantly increased mortalities of 44% and 51.7% in the first 48 h after delivery and transfer^29^, ^37^ (Table 3).

**Table 3:** Neonatal clinical outcomes.

Study ID (year)	Mortality	Hypothermia	Respiratory distress	Hypoglycemic	Cardiovascular stability
1. Roux et al.^24^ (1989) South Africa	None reported	7 neonates (pretransfer) and 11 neonates (posttransfer)—all in private ambulances had temperatures outside the normal 36–37°°C	5 out of 6 preterms	3 neonates—all in private ambulances had a blood glucose of 1.33–2.5 mmol/L pretransport and posttransport	13 neonates required. >30% oxygen during the transfer
2. Njokanma and Fagbule^29^ (1994) Nigeria	63.5% 73.7% in <1.5 kg 54.6% in >1.5 kg 65% in <37 weeks 16.7% >37 weeks	Temperature <35°C Preterm 27.5% % Term 8.7%	26% of preterm	7.5% of preterm 4.3% of term	Not analyzed
3. Pieper et al.^28^ (1994) South Africa	21%	Not analyzed	RDS 7 Meconium aspiration 7, pneumonia 6, peumothorax 4	Not analyzed	Not analyzed
4. Ndiaye et al.^36^ (2003) Senegal	Not analyzed	Not analyzed	Not analyzed	Not analyzed	Not analyzed
5. Enweronu‐Laryea et al.^34^ (2008) Ghana	Survival of out‐born newborns <2.5 kg improved from 56% (before) to 68% (after)	Not analyzed	Not analyzed	Not analyzed	Not analyzed
6. Dicko et al.^41^ (2010) Mali	32%	759/760, 99.9% had temperatures, <37° C	13.9% Birth asphyxia 24%	Not analyzed	Not analyzed
7. De Vries et al.^25^ (2011) South Africa	Not analyzed	Not analyzed	Not analyzed	Not analyzed	Not analyzed
8. Nalwadda et al.^42^ (2013) Uganda	Not analyzed	Not analyzed	Not analyzed	Not analyzed	Not analyzed
9. Nsibande et al.^27^ (2013). South Africa	Not analyzed	Not analyzed	Not analyzed	Not analyzed	Not analyzed
10. Katamea et al.^39^ (2014) Congo	27% *Gestational age* <37 weeks 37.5% ≥37 weeks 15.7%	Infection in 49.3% Temperature instability not analyzed	Not analyzed	Not analyzed	Not analyzed
11. Nlend et al.^38^ (2016) Cameroon	15/73 (20.5%)	20% had temp <36°C The mean temperature in the dead infants upon their arrival to the hospital was 35.5°	14/73 (19%)	Not analyzed	Not analyzed
12. Abdulraheem et al.^30^ (2016) and Tongo et al.^31^ (2020) Nigeria	4.7% certified dead‐on arrival 15% died within 24 h of presentation 3.3% died between 24 and 48 h of presentation	5.1%	Apnea 9%	12.5%	Hypoxia 28.4% Acidosis 8.2% Unrecordable BP 16.5% The median TRIP score on arrival was 17 with 23% having a very severe score of >30 severe 19.4%, moderate 24.6%, and low 33%
13. Ashokcoomar and Naidoo^43^ (2016) South Africa	Not analyzed	Not analyzed	Apnea 1.7% respiratory distress syndrome 40.8%	Not analyzed	Not analyzed
14. Faye et al.^37^ (2016) Senegal	22.3% (29 deaths) 3 newborns were dead on arrival and 6 were in cardiorespiratory arrest 51.7% occurred during the first 48 h of hospitalization	33.8%	Present in 50% on arrival	23.8%	18.1%
15. Accorsi et al.^33^ (2017) Ethiopia	Not analyzed	Not analyzed	Not analyzed	Not analyzed	Not analyzed
16. Sory et al.^40^ (2019) Guinea	46.8% 95.7% in <1 kg, 39.8% 1–1.5 kg, 44.23% in >1.5 kg. *Gestational age* <28 weeks, 28% 28–32 weeks 58% 32–37 weeks 11.9% >37 weeks 1.7% *Gender* Male: 49.7% Female: 40.3%	Not analyzed	Apnea/respiratory distress in 18%	Not analyzed	Not analyzed
17. Teklu et al.^32^ (2020) Ethiopia	Not analyzed	Not analyzed	Not analyzed	Not analyzed	Not analyzed
18. Tette et al.^35^ (2020) Ghana	7.8%	49% on arrival	Abnormal breathing patterns in 62.5%	Not analyzed	On arrival, 70.2% survivors had a normal heart rate 41.7% of those who died had normal heart rates.
19. Okonkwo et al.^12^ Nigeria	Overall mortality 26.1% Preterm mortality 37.8%	<35°C 4.4% 35–37.5°C 62.6%	Gasping 1.8% <30 breaths/min 1.8% >60 breaths/min 40%	<40 mg/dL 7.7% 40–150 mg/dL 80.7%	Cyanosis 23% Hypoxia 38.3% Tachycardia 13.9%

Abbreviation: BP, blood pressure.

In relating mortality to mode of transport, one study reported an increased mortality in neonates transported by bus as compared to taxi^35^ and one study reported 100% survival of babies transported by fixed‐wing aircraft, compared to 70% survival if neonates transported by specially equipped ground ambulance.^28^ Two studies on the compliance and effectiveness of newborn referrals made by community health workers^27^, ^42^ into health facilities demonstrated high compliance and a pivotal role of community health workers in the neonatal transfer process. One study reported on the quality of interhospital ambulance neonatal transfers^24^ and another study focused on barriers to the effective functioning of referral systems for newborns,^32^ reported a lack of ambulance, uncoordinated referral and communication patterns, family reluctance, and patient costs as the major aggravating factors to neonatal transfer. Five studies examined transport modalities (means of transport, the reason for transfer, transfer delay/detours, and distances traveled)^12^, ^28^, ^35^, ^38^, ^43^ and reported erratic and uncoordinated transfer itinerary, increased prereferral facility transfer times, prolonged transfer times with associated high mortality in transferred neonates.

One study assessed the cost‐effectiveness of a dedicated obstetric and neonatal transport service from rural areas to an established emergency obstetric and neonatal care service and reported improved neonatal outcomes associated with improved communication and a highly cost‐effective service.^33^

Studies were not uniform in reporting neonatal outcomes—hypoglycemic, hypothermia, respiratory distress, and cardiovascular instability associated with the transport process aside from mortality. The studies that reported on these revealed poor outcomes. Nine studies reported on hypothermia in transported neonates albeit having differing definitions of hypothermia. Dicko et al. reported that 99% of the study population had a temperature of <37°C,^41^ with the other studies reporting abnormal temperatures in 20%–67% of transported neonates.^12^, ^29^, ^35^, ^37^, ^38^, ^39^

## 4 DISCUSSION

This review summarizes the evidence to date on the transport of neonates in SSA. It demonstrates huge deficiencies in all the critical aspects of the neonatal transport service and a lack of adherence to established standards of transport. Infants delivered in the community or lower‐level facilities continue to face uncertain journeys to higher centers of care with unacceptable high morbidities and mortalities.

A child born in SSA with a NMR of 27 deaths per 1000 live births in 2022, is 11 times more likely to die in the first month of life than a child born in the developed world. This is followed by Central and Southern Asia at 22 deaths per 1000 live births.^46^

With current high levels of neonatal mortality and only modest trends in reducing neonatal mortality in SSA, it is unsurprising that estimates by United Nations International Children's Emergency Fund project 42 of 48 countries in the region to miss the SDG neonatal mortality target by 2030. This emphasizes the need to urgently examine ways to reduce NMRs in the region hand in hand with a clear awareness of local contextual realities.^47^, ^48^

This review found neonatal transfers in the whole subregion to be disorganized. Several studies highlighted the nonexistence of or lack of adherence to standardized pathways for the transport of sick newborns. Most transfers were effected without standard prestabilization with poor communication between referring facilities. The main ethos of neonatal transfer is to keep the neonate stable and monitored during transfer and to forestall further deterioration on arrival at the receiving hospital. Most neonates from these studies were transported in the arms of parents or family members, on public transport with no monitoring and consequently experienced disproportionately poor outcomes at the receiving facilities. Where transportation was available, there remained huge concerns about the preparedness/training as well as the adequacy of accompanying health workers. An organized neonatal transport service centered around an organized specialized retrieval team has been associated with improved outcomes in other studies.^47^, ^48^ For the SSA region, there is an urgent need to improve or implement district or regionalized neonatal referral and transport systems with a capacity to harness cost‐effective measures to meet the demands of the health system.

Another critical piece of evidence was the acute inadequacy of relevant basic equipment where transport was available,^24^, ^30^, ^43^ the absence of equipment in privately adapted ambulances,^33^ or state ambulances,^12^ as well as a lack of use of evidence‐based cost‐effective methods of thermal stability.

Improving the conditions of transported sick neonates should include the prevention of hypothermia by ensuring the availability of incubators, radiant heaters, heated water‐filled mattresses, and KMC.^49^ KMC has been recommended in a Cochrane review as an effective and safe alternative to conventional neonatal care for LBW infants, mainly in resource‐limited countries.^50^ A study by Sontheimer in 2004 reported that infants (preterm *n* = 20; term *n* = 11) transported in a KMC position were physiologically stable and maintained a normal body temperature during ground ambulance transport.^51^ In the context of limited resources settings such as SSA, the unique benefits of KMC being an inexpensive method of maintaining physiologic stability during transport and also fostering parent‐infant bonding and breastfeeding can be harnessed.

An improvement in the overall neonatal outcomes in the subregion demands a critical understanding of the dynamics of the entire transfer process within the context of the resources in the subregion. This starts from the delivery of the neonate through to the decision to transfer, the transfer itself, and feedback communication to the referring facility. Evidence from this review reveals a lack of adequate human resources at all levels of this process. Reassuringly, the studies on the role of community health workers in the neonatal care and transfer process were significant. This reinforces the need to improve training and skills in neonatal stabilization and early referral by this cadre of health workers. They could also hold an answer to circumvent some of the major barriers to the transfer process in the subregion (family reluctance and patient costs related to referral) as identified by Teklu et al.^32^

Finally, 7 years after the only systematic review in developing countries was published,^11^ and halfway into the timeline for the achievement of the SDG 3 target, SSA still has a disproportionately high NMR and a dearth of well‐planned studies on neonatal transport. The lack of randomized controlled studies makes the formulation of context‐specific recommendations challenging. Available studies however reveal a pervasive unstructured, minimally resourced, and poorly monitored neonatal transport service with poor outcomes for transported neonates. The lack of dedicated neonatal transport teams and the heavy reliance on public transport, yet the lack of use of evidenced‐based cost‐effective measures of thermal stability impacts heavily on morbidity and mortality in most studies.

## 5 CONCLUSION

The neonatal transport process in SSA reveals widespread inefficiencies, lack of use of standard referral pathways, and yet lack of evidence on the use of low‐cost interventions in such a resource‐constrained setting.

Strengthening the human resource capacity at all levels of care including the neonatal transport service, improved training and empowerment of the community health workers, effective triage and initial stabilization, and communication with referral centers may hold the key to improving the survival of transported neonates in the subregion. Large‐scale implementation science evaluations for supplying appropriate transfer modes with low‐cost evidence‐based interventions such as KMC are required to assess the short‐ and long‐term impact of improved neonatal transport on neonatal mortality and morbidity.

## 6 STRENGTHS AND LIMITATIONS

This study summarizes to‐date evidence from studies in SSA that have explored enabling or deterrent activities in the neonatal transport process. It is the only systematic review that focuses on SSA as a region in LMICs with unique economic challenges and therefore provides easily available evidence for researchers from the subregion. All the steps for conducting and reporting systematic reviews were rigorously adhered to including registration of the study protocol. Our results can serve as a guide for designing and reporting future studies in this field.

There are however many potential limitations. First, only peer‐reviewed articles were included, and no search of the gray literature was conducted which may mean that additional relevant studies might have been missed. The wide heterogeneity in the included primary studies, particularly in sample size, design, definitions, and focus made it challenging for the pooling of results to enable far‐reaching conclusions. The lack of well‐designed studies particularly randomized control trials in this field in the subregion potentially has significant effects on the quality of the included studies.

## AUTHOR CONTRIBUTIONS

**Emmanuel Okai**: Conceptualization; data curation; formal analysis; methodology; writing—original draft; writing—review and editing. **Frankie Fair**: Data curation; formal analysis; methodology; supervision; writing—review and editing. **Hora Soltani**: Data curation; formal analysis; supervision; writing—review and editing.

## CONFLICT OF INTEREST STATEMENT

The authors declare no conflict of interest.

## ETHICS STATEMENT

This article has been written in accordance with Wiley's Best Practice Guidelines on Publishing Ethics with no research misconduct. Approval by a research ethics committee was not required for this study since the review only included published and publicly accessible data. Informed consent, written or verbal was not required for this study.

## TRANSPARENCY STATEMENT

The lead author Emmanuel Okai affirms that this manuscript is an honest, accurate, and transparent account of the study being reported; that no important aspects of the study have been omitted; and that any discrepancies from the study as planned (and, if relevant, registered) have been explained.

## Supporting information

Supporting information.

Supporting information.

## Data Availability

The data that support the findings of this study are available in the supplementary material of this article.

## References

[1] You D , Hug L , Ejdemyr S , et al. Global, regional, and national levels and trends in under‐5 mortality between 1990 and 2015, with scenario‐based projections to 2030: a systematic analysis by the UN inter‐agency group for child mortality estimation. Lancet. 2015;386(10010):2275‐2286. 26361942 10.1016/S0140-6736(15)00120-8

[2] UNICEF Data . Levels and Trends in Child Mortality. UNICEF; 2015.

[3] Bhutta ZA , Das JK , Bahl R , et al. Can available interventions end preventable deaths in mothers, newborn babies, and stillbirths, and at what cost? Lancet. 2014;384(9940):347‐370. 24853604 10.1016/S0140-6736(14)60792-3

[4] Kumar S , Kumar N , Vivekadhish S . Millennium development goals (MDGS) to sustainable development goals (SDGS): addressing unfinished agenda and strengthening sustainable development and partnership. Indian J Community Med. 2016;41(1):1‐4. 26917865 10.4103/0970-0218.170955 PMC4746946

[5] Sharrow D , Hug L , You D , et al. Global, regional, and national trends in under‐5 mortality between 1990 and 2019 with scenario‐based projections until 2030: a systematic analysis by the UN inter‐agency group for child mortality estimation. Lancet Global Health. 2022;10(2):e195‐e206. 35063111 10.1016/S2214-109X(21)00515-5 PMC8789561

[6] The World Bank . World Bank country and lending groups—World Bank data help desk. Accessed February 22, 2023. https://datahelpdesk.worldbank.org/knowledgebase/articles/906519

[7] Statista . Sub‐Saharan Africa—total population 2011‐2021. Accessed March 6, 2023. https://www.statista.com/statistics/805605/total-population-sub-saharan-africa/

[8] World Bank Group . Where the extreme poor live. Accessed March 31, 2021. https://blogs.worldbank.org/opendata/where-extreme-poor-live

[9] Rosa‐Mangeret F , Benski AC , Golaz A , et al. 2.5 million annual Deaths—are neonates in low‐ and middle‐income countries too small to be seen? A bottom‐up overview on neonatal morbi‐mortality. Trop Med Infect Dis. 2022;7(5):64. 35622691 10.3390/tropicalmed7050064 PMC9148074

[10] Horbar JD , Badger GJ , Carpenter JH , et al. Trends in mortality and morbidity for very low birth weight infants, 1991‐1999. Pediatrics. 2002;110(1):143‐151. 12093960 10.1542/peds.110.1.143

[11] Niermeyer S , Domek G . Neonatal Transport in Developing Country Settings: A Systematic Review. Pan American Health Organization; 2016.

[12] Okonkwo IR , Imuetinyan abhulimhen‐Iyoha B , Anene Okolo A . Newborn transport practices: influence on newborn survival in Benin City, Nigeria. Am J Pediatr. 2020;6(3):346‐352.

[13] Karim AM , Admassu K , Schellenberg J , et al. Effect of Ethiopia's health extension program on maternal and newborn health care practices in 101 rural districts: a dose‐response study. PloS one. 2013;8(6):e65160. 23750240 10.1371/journal.pone.0065160 PMC3672192

[14] World Health Organization . Kangaroo Mother Care: A Practical Guide. Department of Reproductive Health and Research, World Health Organization; 2003.

[15] Lawn JE , Mwansa‐Kambafwile J , Horta BL , Barros FC , Cousens S . Kangaroo mother care' to prevent neonatal deaths due to preterm birth complications. Int J Epidemiol. 2010;39(suppl 1):i144‐i154. 20348117 10.1093/ije/dyq031 PMC2845870

[16] Moore ER , Bergman N , Anderson GC , Medley N . Early skin‐to‐skin contact for mothers and their healthy newborn infants. Cochrane Database Syst Rev. 2016;11:CD003519. 27885658 10.1002/14651858.CD003519.pub4 PMC6464366

[17] Johnston C , Campbell‐Yeo M , Disher T , et al. Skin‐to‐skin care for procedural pain in neonates. Cochrane Database Syst Rev. 2017;2:CD008435. 28205208 10.1002/14651858.CD008435.pub3 PMC6464258

[18] Page MJ , McKenzie JE , Bossuyt PM , et al. The PRISMA 2020 statement: an updated guideline for reporting systematic reviews. Int J Surg. 2021;88:105906. 33789826 10.1016/j.ijsu.2021.105906

[19] Hong QN , Pluye P , Fàbregues S , et al. Mixed Methods Appraisal Tool (MMAT), version 2018. Registration of Copyright (#1148552). Canadian Intellectual Property Office, Industry Canada.; 2018.

[20] Popay J , Roberts H , Sowden A , et al. Guidance on the Conduct of Narrative Synthesis in Systematic Reviews: A Product From the ESRC Methods Programme Version. Vol 1. Lancaster University; 2006:b92.

[21] Mmbaga BT , Lie RT , Kibiki GS , Olomi R , Kvåle G , Daltveit AK . Transfer of newborns to neonatal care unit: a registry‐based study in Northern Tanzania. BMC Pregnancy Childbirth. 2011;11(1):68. 21970789 10.1186/1471-2393-11-68 PMC3206461

[22] Tayler‐Smith K , Zachariah R , Manzi M , et al. An ambulance referral network improves access to emergency obstetric and neonatal care in a district of rural Burundi with high maternal mortality. Trop Med Int Health. 2013;18(8):993‐1001. 23682859 10.1111/tmi.12121

[23] Moher D , Liberati A , Tetzlaff J , Altman DG , for the PRISMA Group . Preferred reporting items for systematic reviews and meta‐analyses: the PRISMA statement. BMJ. 2009;339:b2535. 19622551 10.1136/bmj.b2535 PMC2714657

[24] Roux JC , Nolte AG , Muller ME . Study of the quality of interhospital transport of sick neonates by selected ambulances in the Witwatersrand area. Curationis. 1989;12(3‐4):34‐37. 2632099

[25] De Vries S , Wallis LA , Maritz D . A retrospective evaluation of the impact of a dedicated obstetric and neonatal transport service on transport times within an urban setting. Int J Emerg Med. 2011;4(1):28. 21672232 10.1186/1865-1380-4-28 PMC3131248

[26] Ashokcoomar P , Naidoo R . An analysis of inter‐healthcare facility transfer of neonates within the eThekwini health district of KwaZulu‐Natal. Afr J Emergency Med. 2013;3:S4. 10.1016/J.AFJEM.2013.08.006 27138674

[27] Nsibande D , Doherty T , Ijumba P , et al. Assessment of the uptake of neonatal and young infant referrals by community health workers to public health facilities in an urban informal settlement, KwaZulu‐Natal, South Africa. BMC Health Serv Res. 2013;13(1):47. 23388385 10.1186/1472-6963-13-47 PMC3579691

[28] Pieper CH , Smith J , Kirsten GF , Malan P . The transport of neonates to an intensive care unit. S Afr Med J. 1994;84(11 suppl):801‐803. 8914542

[29] Njokanma F , Fagbule D . Outcome of referred neonates weighing less than 2500 g. Trop Geogr Med. 1994;46(3):172‐174. 7941010

[30] Abdulraheem MA , Tongo OO , Orimadegun AE , Akinbami OF . Neonatal transport practices in Ibadan, Nigeria. Pan Afr Med J. 2016;24:216. 27800071 10.11604/pamj.2016.24.216.8651 PMC5075447

[31] Olukemi T , Abdulraheem MA , Orimadegun AE , Felix OA . Immediate outcomes of neonatal transport in a tertiary hospital in South‐west of Nigeria. Global J. Pediatr Neonatal Care. 2020;2:1‐6. 10.33552/GJPNC.2020.02.000529

[32] Teklu AM , Litch JA , Tesfahun A , et al. Referral systems for preterm, low birth weight, and sick newborns in Ethiopia: a qualitative assessment. BMC Pediatr. 2020;20(1):409. 32861246 10.1186/s12887-020-02311-6 PMC7456368

[33] Accorsi S , Somigliana E , Solomon H , et al. Cost‐effectiveness of an ambulance‐based referral system for emergency obstetrical and neonatal care in rural Ethiopia. BMC Pregnancy Childbirth. 2017;17(1):220. 28701153 10.1186/s12884-017-1403-8 PMC5506594

[34] Enweronu‐Laryea CC , Nkyekyer K , Rodrigues OP . The impact of improved neonatal intensive care facilities on referral pattern and outcome at a teaching hospital in Ghana. J Perinatol. 2008;28(8):561‐565. 18563167 10.1038/jp.2008.61

[35] Tette EMA , Nuertey BD , Akaateba D , Gandau NB . The transport and outcome of sick outborn neonates admitted to a regional and district hospital in the upper west region of Ghana: a cross‐sectional study. Children. 2020;7(3):22. 32244943 10.3390/children7030022 PMC7140801

[36] Ndiaye O , Diallo D , Diouf S , et al. Neonatal mortality associated with transfer of low birth weight newborns. Assessment of a neonatal care unit of Dakar. Dakar Med. 2003;48(1):7‐11. 15776642

[37] Faye PM , Dieng YJ , Diagne‐Guèye NR , et al. Problématique des transferts néonatals dans la région de Dakar (Sénégal). Revue de médecine périnatale. 2016;8(2):94‐102.

[38] Nlend AE , Zeudja C , Nsoa L . Transfer and transport of newborn babies in vital distress in yaoundé, Cameroon: situational analysis conducted in a reference hospital. Pan Afr Med J. 2016;25:214. 28270906 10.11604/pamj.2016.25.214.9642 PMC5326266

[39] Katamea T , Mukuku O , Kamona L , et al. Mortality risk factors in newborns transferred to the neonatal unit of the Hospital Jason Sendwe Lubumbashi, DR Congo. Pan Afr Med J. 2014;19:169. 25810805 10.11604/pamj.2014.19.169.4018 PMC4364683

[40] Sory DI , Sory D , N'fanly C , et al. Neonatal mortality associated with the referral of low birth weight newborns to the Institute of Child Nutrition and Health (INSE). Open J Pediatr. 2019;09(4):287‐295.

[41] Dicko TF , Sylla M , Diakité AA , et al. Problems of neonatal transfer to the pediatric service of the CHU Gabriel touré of Bamako. Mali Med. 2010;25(4):25‐28. 21470952

[42] Nalwadda CK , Waiswa P , Kiguli J , et al. High compliance with newborn community‐to‐facility referral in eastern Uganda: an opportunity to improve newborn survival. PloS one. 2013;8(11):e81610. 24312326 10.1371/journal.pone.0081610 PMC3843697

[43] Ashokcoomar P , Naidoo R . An analysis of inter‐healthcare facility transfer of neonates within the eThekwini health district of KwaZulu‐natal, South Africa. S Afr Med J. 2016;106(5):514‐518. 27138674 10.7196/SAMJ.2016.v106i5.8554

[44] Ravikumar SP , Kaliyan A , Jeganathan S , Manjunathan R . Post‐transport TOPS score as a predictive marker of mortality among transported neonates and its comparative analysis with SNAP‐II PE. Heliyon. 2022;8(8):e10165. 36033290 10.1016/j.heliyon.2022.e10165 PMC9399961

[45] Morse S , Groer M , Shelton MM , Maguire D , Ashmeade T . A systematic review: the utility of the revised version of the score for neonatal acute physiology among critically ill neonates. J Perinat Neonatal Nurs. 2015;29(4):315‐344. 26505848 10.1097/JPN.0000000000000135 PMC4624229

[46] United Nations Inter‐agency Group for Child Mortality Estimation (UN IGME). Levels and Trends in Child Mortality (UN IGME), Report 2022. UN IGME; 2023.

[47] Chang AS , Berry A , Jones LJ , Sivasangari S . Specialist teams for neonatal transport to neonatal intensive care units for prevention of morbidity and mortality. Cochrane Database Syst Rev. 2015;2015(10):CD007485. 26508087 10.1002/14651858.CD007485.pub2 PMC9239562

[48] Belway D , Henderson W , Keenan SP , Levy AR , Dodek P . Do specialist transport personnel improve hospital outcome in critically ill patients transferred to higher centers? A systematic review. J Crit Care. 2006;21(1):8‐17. 16616617 10.1016/j.jcrc.2005.12.008

[49] Sundrani EJ , Katariya U , Mulye S , Yadav D , Patel DS , Sundrani O . Effect of current neonatal transport services on short‐term outcome of outborn neonates. J Evol Med Dent Sci. 2019;8(1):81‐87.

[50] Conde‐Agudelo A , Belizán JM , Diaz‐Rossello J . Cochrane review: Kangaroo mother care to reduce morbidity and mortality in low birthweight infants. Evidence‐Based Child Health Cochrane Rev J. 2012;7(2):760‐876. 10.1002/14651858.CD002771 12804436

[51] Sontheimer D , Fischer CB , Buch KE . Kangaroo transport instead of incubator transport. Pediatrics. 2004;113(4):920‐923. 15060247 10.1542/peds.113.4.920

