# Supplementary material for: Neonatal transport practices and effectiveness of the use of low‐cost interventions on outcomes of transported neonates in Sub‐Saharan Africa: A systematic review and narrative synthesis
Source: Health Sci Rep. 2024 Mar 7;7(3):e1938. doi: 10.1002/hsr2.1938 (PMC10918979; doi:10.1002/hsr2.1938)
Supplement: Supplementary file 1 — Supporting information. [file HSR2-7-e1938-s001.docx]

# SEARCH TERMS

A combination of the following keywords would be used:

1. #1 neonat*
2. #2 neonatal
3. #3 preterm
4. #4 prematurity
5. #5 premat*
6. #6 low birth weight
7. #7 small for gestational age
8. #8 (MH " Neonates")
9. #9 (MH " Neonatal Care")
10. #10 (MH "Prematurity")
11. #11 #1 OR #2 OR #3 OR #4 OR #5 OR #6 OR #7 OR #8 OR #9 OR #10 OR #11
12. #12 transport*
13. #13 evacuation
14. #14 retrieval
15. #15 stabilization
16. #16 ambulance
17. #17 car
18. #18 lorry
19. #19 bicycle
20. #20 transport team
21. #21 transport service
22. #22 evacuation team
23. #23 telephone
24. #24 communication
25. #25 Kangaroo mother care
26. #26 KMC
27. #27(MH " Neonatal retrieval")
28. #28 (MH “Neonatal transport”)
29. #29(MH " Patient transfer")
30. #30 (MH “neonatal referral”)
31. #31 #12 OR #13 OR #14 OR #15 OR #16 OR #17 OR #18 OR #19 OR #20 OR #21 OR #22 OR #23 OR #24 OR #25 OR #26 OR #27 OR #28 OR #28 OR #29 OR #3051
32. #32 “neonatal mortality’’
33. #33 “neonatal death**’’**
34. #34 neonatal survival
35. #35 preterm death
36. #35 preterm survival
37. #37 “low birth weight mortality’’
38. #38 “ low birth weight survival’’
39. #39 (MH " neonatal death")
40. #40 (MH " preterm death")
41. #41 # 32 OR # 33 OR # 34 OR #35 OR #35 OR # 37 OR #38 OR 39 OR 40
42. #42 Hypothermia
43. # 43 Hyperthermia
44. #44 temperature
45. #45 “temperature control’’
46. #46 hypoglycaemia
47. #47 blood sugar control
48. #48 hypoxia
49. #49 respiratory distress syndrome
50. #50 hyaline membrane disease
51. # 51 asphyxia
52. #52 hypotension
53. #53 sepsis
54. #54 apnoea
55. #55 #42 OR #43 OR #44 OR #45 OR #46 OR #47 OR #48 OR #49 OR #50 OR #51 OR #52 OR #53 OR #54
56. #56 developing countries
57. #57 low-income countries
58. #58 middle income countries
59. #59 low middle-income countries
60. #60 Africa
61. #65 low human development index countries
62. #66 medium human development index countries
63. #56 #57 OR #58 OR #59 OR #60 OR #61 OR #62
64. #11 #31 AND #41 AND #55 AND #63
65. #11 #31 AND #41 AND #55 AND each of the following countries

Angola, Benin, Botswana, Burkina Faso, Burundi, Cameroon, Cape Verde, Central African Republic, Chad, Comoros, Congo or DRC or democratic republic of Congo, Ivory Coast, Equatorial Guinea, Eritrea, Ethiopia, Gabon, Gambia, Ghana, Guinea, Guinea-Bissau, Kenya, Lesotho, Liberia, Madagascar, Malawi, Mali, Mauritania, Mauritius, Mayotte, Mozambique, Namibia, Niger, Nigeria, Reunion, Rwanda, Saint Helena, Sao Tome and Principe, Senegal, Seychelles, Sierra Leone, Somalia, South Africa, South Sudan, Swaziland, Togo, Uganda, Tanzania, Zambia, Zimbabwe
