# Supplementary material for: Neonatal transport practices and effectiveness of the use of low‐cost interventions on outcomes of transported neonates in Sub‐Saharan Africa: A systematic review and narrative synthesis
Source: Health Sci Rep. 2024 Mar 7;7(3):e1938. doi: 10.1002/hsr2.1938 (PMC10918979; doi:10.1002/hsr2.1938)
Supplement: Supplementary file 2 — Supporting information. [file HSR2-7-e1938-s002.docx]

**Neonatal transport practices and effectiveness of the use of low-cost interventions on**

**the morbidity and mortality of transported neonates in Sub-Saharan Africa:**

**A systematic review**

Table -- . Definition of methodical quality criteria for Mixed Methods Assessment Tool (sections 1 and 4)

Screening questions

S1. Are there clear research questions?

S2. Do the collected data allow to address the research questions?

Further appraisal may not be feasible or appropriate when the answer is ‘No’ or ‘Can’t tell’ to one or both screening questions.

1. Qualitative

1.1. Is the qualitative approach appropriate to answer the research question?

1.2. Are the qualitative data collection methods adequate to address the research question?

1.3. Are the findings adequately derived from the data?

1.4. Is the interpretation of results sufficiently substantiated by data?

1.5. Is there coherence between qualitative data sources, collection, analysis and interpretation?

4. Quantitative

4.1. Is the sampling strategy relevant to address the research question?

4.2. Is the sample representative of the target population?

4.3. Are the measurements appropriate?

4.4. Is the risk of nonresponse bias low?

4.5. Is the statistical analysis appropriate to answer the research question?

Table 1. **Mixed Methods Appraisal Tools scoring ratings.**

| **Authors (year), Country** | **1.1** | **1.2** | **1.3** | **1.4** | **1.5** |  | **4.1** | **4.2** | **4.3** | **4.4 4.5** | **Score (%)** |
| --- | --- | --- | --- | --- | --- | --- | --- | --- | --- | --- | --- |
| **QUESTION 1** |  |  |  |  |  |  |  |  |  |  |  |
| Qualitative – Patients |  |  |  |  |  |  |  |  |  |  |  |
| Njokanma *et al.* (1994) Nigeria |  |  |  |  |  |  | Y | Y | N | Y N | 60 |
| Nalwadda *et al.* (2013) Uganda |  |  |  |  |  |  | Y | Y | Y | Y Y | 100 |
| Roux et al (1989) South Africa |  |  |  |  |  |  | Y | Y | Y | Y Y | 100 |
| Dicko et al (2010) Mali |  |  |  |  |  |  | Y | Y | Y | Y Y | 100 |
| Ndiaye et al (2003) Senegal |  |  |  |  |  |  | Y | Y | N | N N | 40 |
| Enweronu-Laryea et al (2008) Ghana |  |  |  |  |  |  | Y | Y | Y | N Y | 80 |
| Teklu et al (2020) Ethiopia | Y | Y | Y | Y | Y |  |  |  |  |  | 100 |
| Nlend et al (2016) Cameroon |  |  |  |  |  |  | Y | N | Y | Y Y | 80 |
| Accorsi et al (2017), Ethiopia |  |  |  |  |  |  | Y | N | N | Y Y | 60 |
| Tette, Edem et al (2020), Ghana |  |  |  |  |  |  | Y | N | Y | Y Y | 80 |
| Okonkwo et al (2020), Nigeria, |  |  |  |  |  |  | Y | Y | Y | Y Y | 100 |
| De Vries (2011), South Africa |  |  |  |  |  |  | Y | Y | Y | Y Y | 100 |
| Abdulraheem et al (2016), Nigeria |  |  |  |  |  |  | Y | Y | Y | Y Y | 100 |
| Ashokcoomar et al (2016), South Africa |  |  |  |  |  |  | Y | N | Y | Y Y | 80 |
| Sory et al (2019), Guinea |  |  |  |  |  |  | Y | Y | Y | Y Y | 100 |
| Faye et al (2016), Senegal |  |  |  |  |  |  | Y | Y | Y | Y Y | 100 |
| Duduzile et al (2013), South Africa | Y | Y | Y | Y | Y |  |  |  |  |  | 100 |
| Katamea et al (2014), Congo |  |  |  |  |  |  | Y | Y | Y | Y Y | 100 |
| Pieper et al (1994), South Africa |  |  |  |  |  |  | Y | Y | N | Y Y | 80 |
